# Effects of Cultivation Modes on Soil Protistan Communities and Its Associations with Production Quality in Lemon Farmlands

**DOI:** 10.3390/plants14132024

**Published:** 2025-07-02

**Authors:** Haoqiang Liu, Hongjun Li, Zhuchun Peng, Sichen Li, Chun Ran

**Affiliations:** 1 Citrus Research Institute, Southwest University, Beipei District, Chongqing 400715, China; 2 National Engineering Research Center for Citrus, Chinese Academy of Agricultural Sciences, Beipei District, Chongqing 400712, China

**Keywords:** greenhouse, open-field cultivation, nutrient cycling, protists, community assembly mechanism, biochemical properties of the soil, fruit quality

## Abstract

Citrus is one of the most widely consumed fruits in the world, and its cultivation industry continues to develop rapidly. However, the roles of soil protistan communities during citrus growth are not yet fully understood, despite the potential significance of these communities to the health and quality of citrus. In this study, we examined the soil properties and protistan communities in Eureka lemon farmlands located in Chongqing, China, during the flowering and fruiting stages of cultivation, both in greenhouse and open-field settings. In general, the majority of the measured soil properties (including nutrients and enzyme activities) exhibited higher values in open-field farmlands in comparison to those observed in greenhouse counterparts. According to the results of high-throughput sequencing based on the V9 region of eukaryotic 18S rRNA gene, the diversity of soil protistan communities was also higher in open-field farmlands, and both lemon growth stage and cultivation modes showed significant effects on soil protistan compositions. The transition from traditional agricultural practices to greenhouse farming resulted in a significant transformation of the soil protistan community. This transformation manifested as a shift towards a state characterized by diminished nutrient cycling capabilities. This decline was evidenced by an increase in phototrophs (Archaeplastida) and a concomitant decrease in consumers (Stramenopiles and Alveolata). Community assembly analysis revealed deterministic processes that controlled the succession of soil protistan communities in lemon farmlands. It has been established that environmental associations have the capacity to recognize nitrogen in soils, thereby providing a deterministic selection process for protistan community assembly. Furthermore, a production index was calculated based on 12 quality parameters of lemons, and the results indicated that lemons from greenhouse farms exhibited a lower quality compared to those from open fields. The structure equation model revealed a direct correlation between the quality of lemons and the cultivation methods employed, as well as the composition of soil protists. The present study offers insights into the mechanisms underlying the correlations between the soil protistan community and lemon quality in response to changes in the cultivation modes.

## 1. Introduction

Agricultural ecosystems have provided humans with food, forage, bioenergy, and pharmaceuticals, which are essential for human well-being [1]. It is important to note that there are usually multiple planting modes for the same crop, such as greenhouse vs. natural growth [2]. Furthermore, agricultural land designated for the cultivation of the same crop may exhibit divergent geographical characteristics due to limitations imposed by the topography, such as plains as opposed to hills [3]. Furthermore, the practice of rotation is a prevalent cultivation method, and the conditions experienced by corps in rotation can vary significantly, including the contrast between paddy and dry fields [4]. It has been demonstrated that these variations within agricultural ecosystems have the capacity to influence the soil properties and regulate the crop quality. A case in point is that of rice-wheat rotation, a highly intensive agricultural planting mode in China. It has been demonstrated that this practice enhances soil fertility and productivity, a consequence of the effects of straw return [5]. A study of rice paddy fields employing diverse planting techniques also identified significant variations in soil bulk density, tiller number, spikelet number per panicle, panicle length, panicle weight, and grain yields [6]. Furthermore, analysis of pear orchards within the river basin revealed a decline in soil nutrient levels, while concurrently demonstrating enhanced yield in comparison to conventional natural orchards [7]. Consequently, farmlands that cultivate the same crop but employ diverse cultivation techniques or land backgrounds should be the focus of extensive investigation to develop optimized management.

In addition to the soil properties, it should be noted that soil microbial communities of agricultural ecosystems will also change under different planting practices [8]. However, the majority of previous studies have primarily focused on the effects of different planting modes on the soil bacterial communities in agricultural ecosystems [9,10,11]. In recent years, the significant roles of protists in the promotion of plant growth and enhancement of plant health have come to the forefront of research [12]. A study of agricultural ecosystems across Europe indicated that land use and management practices are significant factors influencing soil protistan communities [13]. Another study demonstrated that soil protists can function as indicators and determinants of plant performance, thereby stimulating plant performance through microbiome interactions [14]. Furthermore, the potential of protists in biotechnological applications aimed at reducing pesticide and fertilizer usage in sustainable agriculture has been explored [15]. Despite the existence of some studies on the subject, there remains a paucity of precise information regarding soil protistan communities and their relationships with the crop quality in agricultural ecosystems.

Citrus fruits are among the most widely traded commodities on the global market, owing to their refreshing flavor and high vitamin C content [16]. Among the diverse range of citrus species, the Eureka lemon is a popular citrus tree that year-round produces an abundance of fine, market-quality fruit with tart juice and flavourful zesty peel [17]. China is a major producer of lemons on a global scale, with Eureka lemons being a prominent variety cultivated in the country. The production of these lemons is consistent throughout the year in regions such as Chongqing and Sichuan Province. It is hypothesized that the cultivation modes and their variations may have an effect on the quality of lemons, based on the ecological functions of soil protistan communities. In order to further expand the scale of the Eureka lemon industry and increase economic benefits, our study investigated the soil protistan communities on farmlands with different cultivation modes in lemon planting areas of Chongqing Province, China. The objectives of this work were: (i) to portray the soil protistan profiles of lemon farmlands with different cultivation modes; (ii) to dissect the assembly mechanisms of soil protistan communities and their associations with soil properties; (iii) to explore the relationships of lemon quality with cultivation modes, soil properties, and soil protistan communities.

## 2. Materials and Methods

### 2.1. Experience Scheme and Sample Collection

Soil samples were collected from the agricultural terrain of a lemon farm at the Chongqing Tongnan Seedling Breeding Center located in the Guopo Village, Baizi Town, Chongqing, China. The area under consideration encompasses 16 hectares of citrus farmland, with a history of agricultural activity that spans a period exceeding five years. The collection of soil samples was undertaken in nine farmlands that cultivated the Eureka lemon tree were selected. Among them, three farmlands are orchards with greenhouse (Greenhouse), three farmlands were transformed into orchards from hilly areas (hilly-converted), and three farmlands were transformed into orchards from paddy fields (paddy-converted). Sampling was conducted on the 4th of May, and the 24th of September, 2023, which corresponded to the flowering and fruiting stages of lemon growth, respectively. In a single orchard at the single stage, six trees (a total of 54 trees) were selected to obtain soil samples. For each tree, a multi-point sampling method was employed, which entailed the collection of surface soils at three random points around the lemon trunks (5–10 cm). Each tree was sampled on three occasions to ensure the representation of the data in the final sample. The soil samples were then divided into two parts for the purpose of determining the soil properties. An additional portion of the fresh soil samples was subjected to treatment with liquid nitrogen for the purpose of rapid cooling. Following this, the samples were cryopreserved at −80 °C for the purpose of determining and analyzing the soil protistan communities. A total of 108 soil samples were collected from nine different lemon farms, with six samples being obtained from each farm at each of the lemon growth stages.

### 2.2. Measurements of Soil Properties

Soil moisture content was determined by calculating the proportion of lost weight through a process of oven drying for 12 h at 105 °C. Before measuring the content of soil micronutrients, the soil samples were air-dried. The total nitrogen (TN) of the soil sample was measured using the Kjeldahl method [18], and ammonia, nitrate, and nitrite levels were detected using an Auto Analyzer 3 Digital Colorimeter (Bran & Luebbe Inc., Norderstedt, Germany). Soil pH was measured in a 1:5 soil/water suspension using a pH meter. The total phosphorus (TP) was measured using NaOH digestion and the molybdenum blue colorimetric method [19]. The concentration of available phosphorus (AP) in the soil was determined by the molybdenum antimony colormetric method [20] using an UV-visible spectrophotometer (UV-1200, Mapada, Shanghai, China). The total potassium (TK) and available potassium (AK) of the soil sample were measured using a flame photometer with extracted by NaOH and NH4OAc, respectively [21]. For dissolved organic carbon (DOC), soil oscillated were placed for in an Erlenmeyer, and distilled water was added. Samples were oscillated for 30 min, filtered, and left to rest for 10 min to obtain the leaching solution. Then, the DOC was analyzed using a UV-8000 spectrophotometer (Mapada, Shanghai, China) based on the leaching solution after oxidizing organic carbon with potassium dichromate [22]. The enzyme activities of alkaline phosphatase (ALP), beta-glucosidase (beta-Glu), urease, and cellulase were determined using respective ELISA kits (Shanghai Jiwei Biological Technology Co., Ltd., Shanghai, China) using an RT-6100 spectrophotometer (Rayto Life and Analytical Sciences Co., Ltd., Shenzhen, China). For each measurement of each soil samples, three replicates were performed.

### 2.3. Measurements of Lemon Quality Parameters

A series of lemon quality parameters, including fruit weight, fruit width, fruit height, fruit shape index, pericarp thickness, pericarp weight, valve number, valve weight, seed number, seed weight, solid rate, and edible rate, in each studied farmland were measured after the lemon harvest. All measurements were in accordance with the National Standard of China (GB/T 8210-2011) [23]. Detailed, the weight of whole fruit was measured by an electronic balance. Then, the fruit was disassembled to count its valve number and seed number. Then, weights of pericarp, value, and seed were also measured by an electronic balance. The longitudinal and transverse diameters of lemon were measured by a Vernier caliper, and the fruit shape index was calculated as their ratio. The fruit width, fruit height, and pericarp thickness were also measured by the Vernier caliper. The sold rate was the proportion of the weight of fruit except to the juice with the whole weight of fruit. The edible rate was the proportion of the weight of fruit except seed with the whole weight of fruit.

### 2.4. DNA Extraction and Protistan Community Sequencing

The isolation of microbial DNA from soil samples was accomplished by means of the FastDNA^®^ SPIN kit for soil (MP Biomedicals, Irvine, CA, USA). Agarose gel electrophoresis (1.5%) was performed in order to ascertain the efficacy of the DNA extraction process. The quality of the successfully extracted DNA was then evaluated by NanoDrop ND-1000 Spectrophotometer (NanoDrop, Wilmington, DE, USA). For investigating the protistan communities, the V9 region of the eukaryotic 18S rRNA gene was amplified from the microbial DNA using the primers of 1380F (TCCCTGCCHTTTGTACACAC) and 1510R (CCTTCYGCAGGTTCACCTAC) [24]. The detail processes of PCR and library construction referred to previous studies [25]. Finally, the libraries were subjected to sequencing using the Illumina NovaSeq 6000 platform at BIOZERN Biotech. Co., Ltd. (Shanghai, China).

The assignment of sequenced reads to the corresponding samples was facilitated by the unique barcode at the end of reverse primer for each gene. Subsequently, quality control was performed using the “dada2” package in R v4.2.2 according to the thresholds of Q score > 20 and no ambiguous base [26]. The remaining paired-end reads were then assembled based on the overlap region between them. Subsequently, the assembled reads were clustered into amplicon sequence variants (ASVs), with the chimera was removed using the QIIME2 (Quantitative Insights Into Microbial Ecology 2) program [27]. Taxonomy was assigned to each ASV based on the PR2 v4.12.0 database [28]. Then, ASVs that were assigned into other taxa (Fungi, Metazoa, and Embryophyta) were excluded. Finally, singletons (the total read number of a specific ASV in all samples is 1) were abandoned, and the AVS abundance table was normalized to 18,907 reads before further data analyses.

### 2.5. Statistics Analysis

All data analyses were performed using R v4.2.2, and the results were visualized by the “ggplot2” package. Four alpha-diversity indices, Chao1 (richness), Pd_faith (evolution), Shannon (diversity), and Pielou_J (evenness), of protistan communities were, respectively, calculated using the “vegan” package v2.6-10. Differences in the soil properties and alpha diversity indices of protistan communities between different cultivation modes and lemon growth stages were examined by Tukey’s HSD test (“multcomp” package v1.4-28). Principal coordinate analysis (PCoA) and PERMANOVA (“vegan” package), based on the Bray–Curtis distance, were performed to demonstrate differences in soil protistan composition from farmlands with different cultivation modes and lemon growth stages. Tukey’s HSD test was also employed to assess the variations in Bray–Curtis distance and dominant protist taxa between different cultivation modes and lemon growth stages. Protists were assigned to three major functional groups, based on their taxonomic affiliation according to three main trophic modes: phototrophic, parasitic, and consumer. The detailed matching information between protist taxonomy and functional groups is referred to in the table previously reported by Singer et al. [29]. Variations in these functional groups between different cultivation modes and lemon growth stages were also assessed by Tukey’s HSD test. PCoA and PERMANOVA were further performed to evaluate variations in the compositions of these three functional groups.

Redundancy analysis (RDA) was performed to evaluate the correlations of soil environmental variables with protistan communities (“vegan” package). Random forest analysis, one of the machine learning method, was constructed based on the soil environmental variables to predict the variations in soil protistan compositions (“RandomForest” package v4.7-1.2). Based on the random forest results, potential key drivers for soil protistan communities were extracted. To explore the community assembly mechanisms, the null model were applied to the protistan communities [30]. In the null model, the phylogenetic distance (betaMNTD) of protistan communities was first calculated and further turns it into the beta nearest taxon index (betaNTI) to distinguish the contribution of deterministic (|betaNTI| > 2) and stochastic (|betaNTI| ≤ 2) processes in community assembly. Differences in the betaMNTD and betaNTI of soil protistan communities between different cultivation modes and lemon growth stages were also tested by Tukey’s HSD test. Furthermore, the Raup–Crick (RC) metric among different protistan communities were also calculated. The classification of community assembly was then compared with the betaNTI, as per the findings of a previous study [31].

Finally, a production index was calculated based on the quality parameters of lemons to evaluate the lemon quality according to the method for soil multifunctional index reported by [32]. Linear regression was used to assess the relationships between the lemon production index and the diversity or composition of soil protistan communities. Then, the direct and indirect effects of cultivation modes on lemon quality mediated by the soil properties and protistan communities were quantified using structural equation modeling (SEM, “lavaan” package v0.6-19).

## 3. Results

### 3.1. Variations in Soil Properties

**Figure 1:**
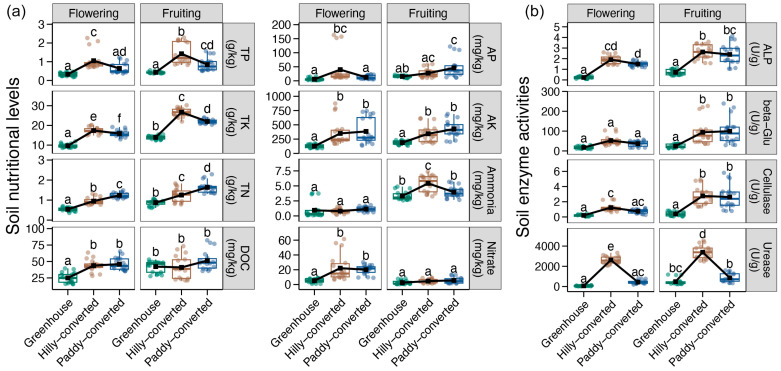
Differences in the soil nutrient levels (**a**) and enzyme activities (**b**) between different cultivation modes and different lemon growth stages. Different lowercases letters in each subfigure represent significant differences between different groups (Tukey’s HSD test, *p* < 0.05).

Here, a total of 14 soil properties from various lemon farmlands at both the flowering and fruiting stages were measured and compared. In the flowering stage, the soil pH was significantly higher in the hilly- and paddy-converted farmlands (alkalescence) compared to that in the greenhouse group (acidescence), while it was similar in all farmlands at the fruiting stage (alkalescence) (Supplementary Figure S1). In contrast, no obvious variation was observed for the soil moisture among all farmlands at the flowering stage, whereas it was significantly higher in the hilly- and paddy-converted farmlands at the fruiting stage (Supplementary Figure S1). With regard to soil nutrients, the highest concentrations of TP and TK were detected in the hilly-converted farmlands at both the flowering and fruiting stages, followed by the paddy-converted farmlands, and the lowest in the greenhouse soils (Figure 1a). Similar results were also evident in the contents of AP and ammonia during the flowering and fruiting stages, respectively (Figure 1a). In addition, the TN concentration at both the flowering and fruiting stages and the AP content at the fruiting stage were shown to be the highest in the paddy-converted farmlands, followed by the hilly-converted samples, and the lowest levels in the greenhouse (Figure 1a). Moreover, we also observed significantly higher concentrations of DOC and AK in the hilly- and paddy-converted farmland compared to those in the greenhouse at both flowering and fruiting stages (Figure 1a). When paying attention to soil enzyme activity, we found significantly higher activities of ALP in the hilly- and paddy-converted farmlands at both flowering and fruiting stages and beta-Glu and Cellulase in the fruiting stage (Figure 1b). Moreover, the activity of urease was found to be significantly higher in the hilly-converted farmlands in comparison to other regions, both at the flowering and fruiting stages (Figure 1b).

### 3.2. Variations in Soil Protistan Diversity

**Figure 2:**
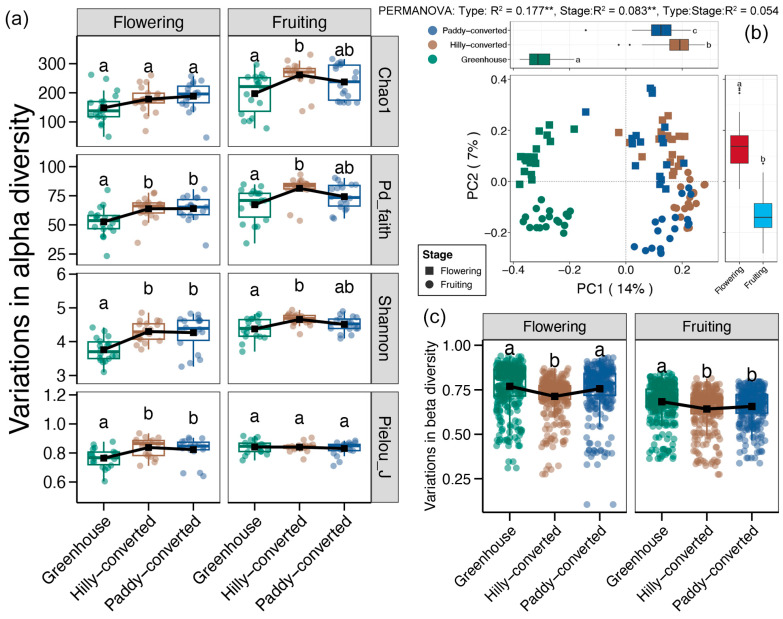
(**a**) Differences in the alpha diversity indices of soil protistan communities between different cultivation modes and different lemon growth stages. (**b**) Principal coordinate analysis (PCoA) and PERMANOVA test based on the Bray–Curtis distance for protistan communities between different cultivation modes and different lemon growth stages. ** represents the significant effects of factors on the variations in protistan communities (adonis test, *p* < 0.01). (**c**) Differences in the Bray–Curtis distance of soil protistan communities between different cultivation modes and different lemon growth stages. Different lowercases letters in each subfigure represent significant differences between different groups (Tukey’s HSD test, *p* < 0.05).

In total, we obtained 3,382,668 high-quality sequences from the 108 soil samples, which were then clustered into 3487 ASVs. According to the taxonomy annotation, a total of 10 phyla, 29 classes, 86 orders, 166 families, 300 genera, and 492 species were identified, with more than 90% and 75% annotated rates at the family and species levels, respectively (Supplementary Figure S2). The rarefaction and species accumulation curves of all groups were tended to be horizontal (Supplementary Figure S3), indicating that the sequencing depth and sampling size could reflect the complete soil protistan communities in lemon farmlands. At the flowering stage, we found significantly higher alpha diversity of soil protistan communities (except the Chao index) in the hilly- and paddy-converted farmlands compared to the greenhouse samples (Figure 2a). In contrast, we observed highest alpha diversity indices of soil protistan communities (except the Pielou_J index) in the hilly-converted farmlands, which were significantly higher than those in greenhouse soils (Figure 2a).

The PCoA, based on the Bray–Curtis distance, showed that soil protistan communities from lemon farmlands, which were cultivated in different ways, were distinctively grouped and distinguished by the PC1 axis (Figure 2b). Moreover, soil protistan communities belonging to disparate lemon growth stages were also differentiated by the PC2 axis (Figure 2b). PERMANOVA revealed that both cultivation mode and growth stage significantly affected the soil protistan communities of lemon farmlands, with higher effects of cultivation modes (*p* < 0.05, Figure 2b). Then, we observed highest variations in soil protistan communities in the greenhouse during the flowering stage, followed by the hilly-converted, and the lowest was found in the paddy-converted farmlands (Figure 2c). During the fruiting stage, variations in soil protistan communities in the hilly-converted farmlands were significantly lower than those in the greenhouse and paddy-converted farmlands (Figure 2c).

### 3.3. Variations in Soil Protistan Compositions and Functions

**Figure 3:**
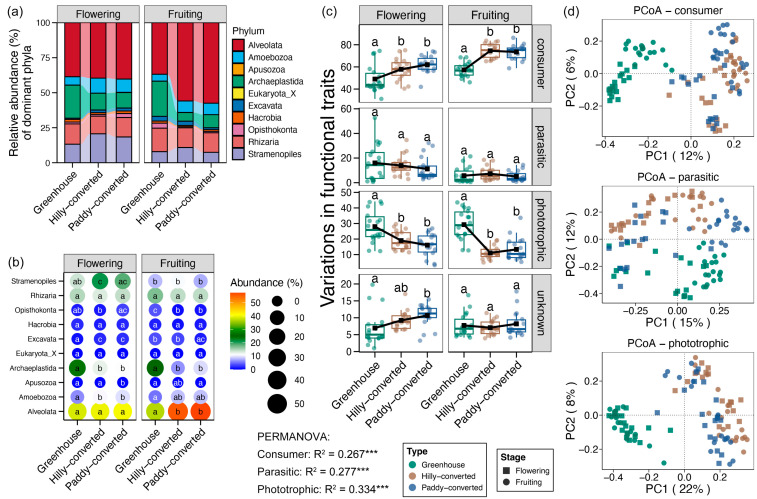
(**a**) Relative abundance (%) of soil protistan phyla among different farmlands. (**b**) Differences in the relative abundance of soil protistan phyla between different cultivation modes and different lemon growth stages. (**c**) Variations in relative abundances of protistan functional traits between different cultivation modes and different lemon growth stages. Different lowercases letters in each subfigure represent significant differences between different groups (Tukey’s HSD test, *p* < 0.05). (**d**) Principal coordinate analysis (PCoA) and PERMANOVA test based on the Bray–Curtis distance for different protistan functional traits between different cultivation modes and different lemon growth stages. *** represents the significant effects of factors on the variations in protistan communities (adonis test, *p* < 0.001).

Alveolata was found as the dominant protistan phylum in lemon farmlands, followed by Archaeplastida, Rhizaria, and Stramenopiles (Figure 3a). The relative abundance of Alveolata was shown to be significantly higher in hilly- and paddy-converted farmlands than that in greenhouse soils at the fruiting stage (Figure 3b). In addition, Archaeplastida was more abundant in greenhouse soils compared to other farmlands during the both flowering and fruiting stages (Figure 3). Moreover, Stramenoplies was enriched in the hilly-converted farmlands at the flowering stage (Figure 3b). At the species level, Pythium was the most abundant, and other main species included Colpoda, Cercomonas, Oxyricha, and Scenedesmus (Supplementary Figure S4). Among them, Oxytricha was enriched in the hilly-converted farmlands at the fruiting stage, and Scenedesmus was more abundant in greenhouse soils at both flowering and fruiting stages (Supplementary Figure S5).

It is evident that protists can be categorized according to their functional traits, which are indicative of their trophic level. The three primary trophic levels of protists are: consumer, parasitic, and phototrophic. In the context of lemon farmlands, a marked increase in the relative abundance of consumers was observed in soils derived from hilly and paddy conversion, both during the flowering and fruiting stages. In contrast, the relative abundance of phototrophic protists exhibited a higher prevalence in greenhouse samples (Figure 3c). In addition, PCoA showed obviously cultivation mode and growth stage were clearly associated with the formation of clusters for consumer and parasitic protists. However, the impact of growth stage on phototrophic protists was found to be comparatively minimal (Figure 3d).

### 3.4. Environmental Assoications and Assembly Mechanisms of Soil Protistan Communities

**Figure 4:**
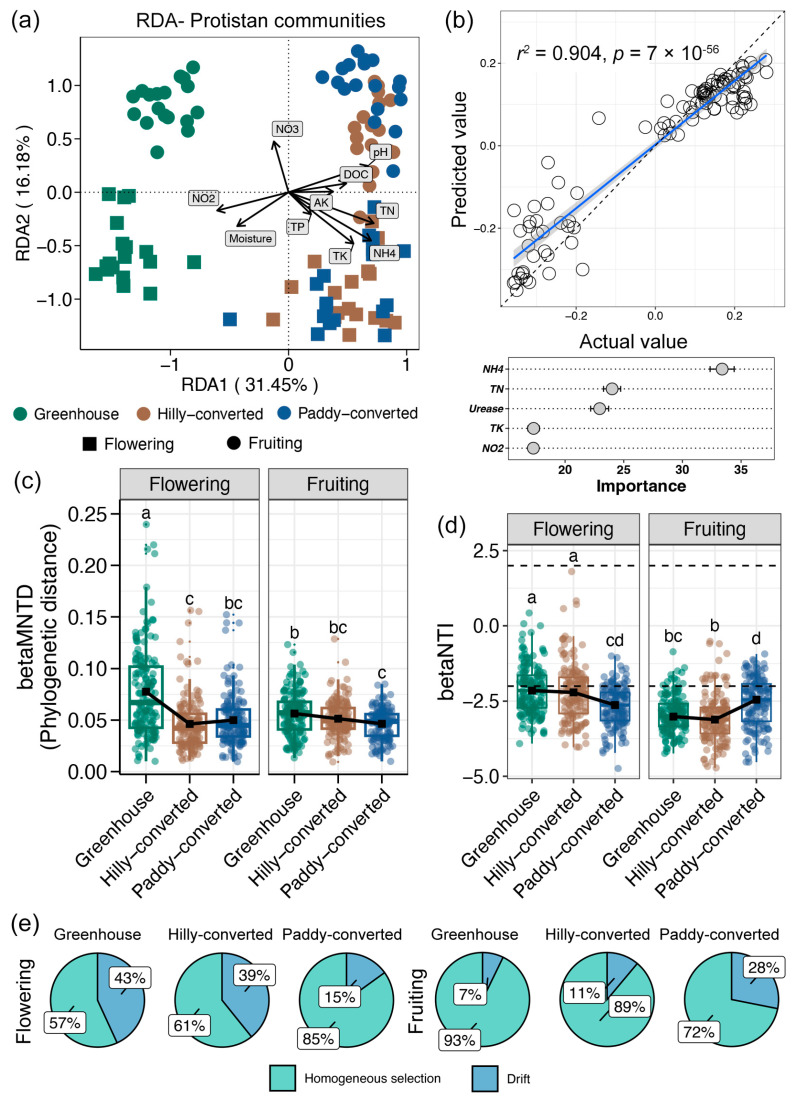
(**a**) Redundancy analysis (RDA) showing the correlations between soil environmental variables with protistan communities in lemon farmlands. (**b**) Linear regression exploring the accuracy of random forest model to predict the variations in soil protistan communities and the key factors for this prediction. Blue line represents the fitted curve and gray shadow represents the 95% confidence interval. Differences in the betaMNTD (**c**) and betaNTI (**d**) of soil protistan communities between different cultivation modes and different lemon growth stages. Different lowercases letters in each subfigure represent significant differences between different groups (Tukey’s HSD test, *p* < 0.05). (**e**) Contributions of different ecological processes for assembly of soil protistan communities.

The RDA was initially employed to assess the correlations between soil environmental variables and protistan communities in lemon farmlands, and the results are shown in Figure 4a. The majority of measured soil environmental variables exhibited significant correlations with the soil protistan communities. Among the factors under consideration, nitrate was the most significant in terms of discrepancies between the stages, with a substantial correlation to the PC2 axis, the axis differentiated protistan communities from the flowering and fruiting stages. To distinguish protistan communities from farmlands with different cultivation modes, ammonia, TK, and TK positively correlated to the hilly- and paddy-converted samples, whereas nitrate and moisture showed positive effects on the greenhouse soils. Furthermore, a random forest model based on soil environmental variables was constructed to predict variations in soil protistan communities, and a more than 90% predicted accuracy was obtained (Figure 4b). Based on the random forest model, ammonia, TN, TK, and nitrite were recognized as the main drivers for variations in soil protistan communities within lemon farmlands (Figure 4b).

Based on the comparison of betaMNTD, we observed significantly higher phylogenetic distances for soil protistan communities in greenhouse compared to other farmlands at both flowering and fruiting stages (Figure 4c). For community assembly, we found the median of betaNTI for all groups were lower than –2, indicating determinism-dominated assembly for soil protistan communities in lemon farmlands (Figure 4d). At the flowering stage, the betaNTI of soil protistan communities at the greenhouse and hilly-converted farmlands were more closed to –2, indicating relatively higher contribution of stochastic processes (Figure 4d). In contrast, the betaNTI was significantly decreased in soil protistan communities at the greenhouse and hilly-converted farmlands at the fruiting stage, showing a more deterministic-controlled community assembly (Figure 4d). The processes of homogeneous selection and drift were recognized as the specific ecological processes for deterministic and stochastic processes, respectively, for soil protistan community assembly in lemon farmlands (Figure 4e). In accordance with the findings of betaNTI, the contribution of heterogeneous selection was about 60% in greenhouse and hilly-converted farmlands during the flowering stage, which increased to 84.97% in paddy-converted farmlands (Figure 4e). Conversely, the contribution of heterogeneous selection increased to approximately 90% in greenhouse and hilly-converted farmlands at the fruiting stage, whereas it decreased to 71.9% in the paddy-converted farmlands (Figure 4e).

### 3.5. Correlations of Soil Protistan Communities and Lemon Quality

**Figure 5:**
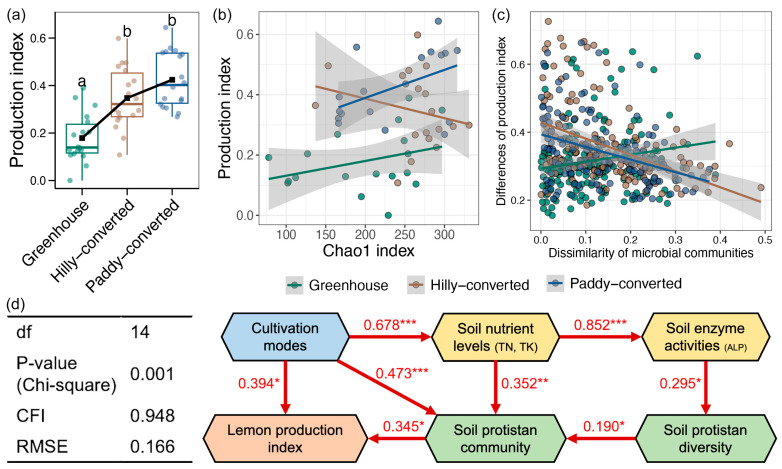
(**a**) Differences in the production index of lemons obtained from different farmcultivation modes. Different lowercases letters represent significant differences between different groups (Tukey’s HSD test, *p* < 0.05). Relationships between lemon production index with soil protistan diversity (**b**) and composition (**c**) in different farmlands. (**d**) Structure equation model showing the direct and indirect effects of farmcultivation modes on lemon quality mediated by soil protistan communities. Only significant relationships were shown in the path diagram, and a red color represents a positive effect. *, **, and *** represent the *p*-value lower than 0.05, 0.01, and 0.001, respectively.

Subsequent to the harvesting of lemons, we measured a series of production indices to evaluate the quality of lemons. Although multiple production indices exhibited variations among farmlands with different cultivation modes, only a significantly higher valve number was observed in lemons from the hilly-converted farmlands (Supplementary Figure S6). Furthermore, we calculated a comprehensive production index based on measured quality parameters, and the value of this index was shown to be significantly higher in lemons from the hilly- and paddy-converted farmlands compared to those from greenhouse (Figure 5a). Then, relationships between the diversity and composition of soil protistan communities with the lemon production index were investigated, respectively. Significant correlations between the soil protistan diversity and the lemon production index were only found in paddy-converted farmlands (Linear regression, *p* < 0.05, Figure 5b). For the relationships between the protistan composition and the lemon production index, significant associations were obtained in hilly- and paddy-converted farmlands (Linear regression, *p* < 0.05, Figure 5c). Finally, an SEM was constructed to explore the mechanisms of variations in lemon quality of farmland type mediated by soil protistan communities (Figure 5d). The production index of lemons was found to be directly influenced by the type of farmland and the soil protistan compositions. The findings of the study demonstrated a direct correlation between variations in soil protistan composition and the farm cultivation modes, soil nutrient levels, and protistan diversity. Soil protistan diversity was directly changed by soil enzyme activities (mainly by ALP), which was directly altered by soil nutrient levels (mainly by TN and TK).

## 4. Discussion

Protists represent a significant group of eukaryotic microorganisms in soils; however, their specific communities and related functions in lemon farmlands have not been extensively documented. The present study was based on a comprehensive sampling of soil protistan communities across three distinct cultivation modes of lemons in Chongqing, China. To the best of our knowledge, the present study is for the first time to provide the soil protistan profiles and explore their variations in lemon farmlands. We observed significant variations in the diversity, composition, and function of soil protistan communities in lemon farmlands with different cultivation modes and distinct lemon growth stages. Moreover, our results showed stronger effects of cultivation modes on soil protistan communities than those of lemon growth stages. Both seasonality and land use have been found to affect the soil protistan communities with different strengths among studies [33,34,35]. In this study, the flowering and fruiting stages of lemons were in spring and autumn, respectively, and the climatic conditions were relatively similar. Conversely, the management of greenhouses is subject to a greater degree of regulation as a consequence of human activities when compared with open-field cultivation. This may be the reason for why this study found that cultivation modes have a stronger impact on soil protistan communities in lemon farmlands.

Evidence has demonstrated that biodiversity exerts a significant influence on a variety of ecosystem functions, including, but not limited to productivity, decomposition, nutrient turnover, and stability [36]. A plethora of studies have demonstrated that an increase in species diversity can enhance ecosystem functions by promoting complementary resource use and influencing particular species traits [37]. Furthermore, biodiversity is essential for providing ecosystem services that support human life, such as food, clean water, disease regulation, climate regulation, and pollination [38]. Therefore, maintaining biodiversity is crucial for sustaining ecosystem functions and services that are vital for both natural systems and human well-being [39]. In our results, we observed significantly lower diversity of soil protistan communities in lemon farmlands with greenhouses compared those in open-air environments (Figure 2a), suggesting the degeneration of soil functions in lands with the greenhouses. Previous studies have revealed the land degeneration in greenhouses due to the soil erosion, salinization, and fertility loss induced by the intensive agricultural practices [40,41]. Moreover, the site-to-site variations in soil protistan communities were also found to be largest in the greenhouse fields (Figure 2c), indicating a weak stability of soil protistan communities in greenhouse farming. It is imperative to acknowledge the significance of a stable soil microbial community in ensuring the consistent execution of ecosystem functions [42]. Thus, our results implied the relatively weak and unstable ecosystem functions of lemon farmlands with greenhouses compared to those in open-air environments.

In contrast to taxonomic composition, the application of functional traits is cumulative in nature, facilitating the evaluation of the roles of microbial communities in ecosystem functions and services [43]. According to the lifestyle, protists can be divided into three functional groups, which are consumers, phototrophs, and parasitic taxa [29]. Among them, the consumer group was the dominant group, followed by parasitic taxa and phototrophs [44], consistent results were also found in our study. More specifically, we found significantly higher relative abundance of phototrophs in lemon fields with greenhouse farming (Figure 3c). This result was consistent with more effective lighting and relatively high temperatures in greenhouses. Most of the phototrophs in lemon farmlands belonged to the Archaeplastida phyla, a group of photoautotrophic organisms encompassed by red algae, green algae, and land plants [45]. These organisms are primary producers, responsible for converting light energy into organic compounds through photosynthesis [46]. However, unlike the importance of phototrophy in marine systems for global carbon fixation and nutrient cycling [47], the standing biomass of phototrophs in soils is negligible as compared with plants, although their turnover is much faster [48]. In contrast to the abundant phototrophs, lemon farmlands with greenhouses showed lower relative abundance of consumers. At the flowering stage, the decreased consumers in greenhouses were mainly Stramenopiles (Figure 3b). Non-photosynthetic Stramenopiles are widespread consumers of bacteria, which play a major role in recycling carbon and nutrients within microbial food webs to maintain the nutrient cycles in the ecosystem [49]. At the fruiting stage, the decreased consumers in greenhouses mainly belonged to the Alveolata phyla, especially Hypotrichia (Figure 3b and Supplementary Figure S5). Hypotrichia are important in nutrient cycling and for the maintenance of soil structure, thus influencing the overall health and productivity of these ecosystems [50]. Based on these information, we could deduce that greenhouses might inhibit nutrient cycling in soil and then damage the ecosystem functions of lemon farmlands.

A determinism (heterogeneous selection)-dominant assembly of soil protistan communities was found in our studied lemon farmlands (Figure 4d,e). This finding suggested that the soil protistan community was mainly governed by environmental filtering [51]. Our results further revealed nitrogen, including TN, nitrate, nitrite, and ammonia, as the key factors to drive the variations in the soil protistan communities of lemon farmlands (Figure 4a,b). Nitrogen has also been found to be a dominant driver for soil protistan community in other agricultural ecosystems, expressed by changes in the protistan community due to nitrogen inputs [52,53]. Moreover, research has shown that protists are more sensitive to nitrogen fertilization than other microorganisms in agricultural soils [54]. These evidence suggest that nitrogen can exert a notable influence on soil protist communities in agricultural systems, potentially leading to changes in their diversity and composition. Given that the crucial roles of soil protists in agricultural ecosystems and changes in soil protist communities due to factors such as nitrogen fertilization can impact nutrient cycling, microbial interactions, and plant health, they ultimately influence the stability and functioning of ecosystems [55,56]. These findings further highlight the importance of understanding and monitoring soil protist communities to assess and manage their implications for ecosystem functioning in the context of environmental changes, including nitrogen inputs.

The primary function of agricultural ecosystems is to produce higher quality agricultural products, and it is the considered opinion of the present author that the ultimate goal of other related research should be related to this fact. Thus, we evaluated the lemon quality of the farmlands involved in our study and its associations with soil properties and protistan communities. Greenhouses have been believed to produce crops with higher yields and consistent seasonality compared to the open-field cultivation as they offer climate control systems [57]. Moreover, greenhouses also help lower the risk of pests and disease transmission, which can lead to increased yields and improved overall product quality [58]. However, it is possible to unknowingly follow practices that can contribute to or directly cause a reduction in crop quality in a greenhouse, such as insufficient venting or poorly maintained exhaust fans [59]. In our results, we observed a substantial reduction in lemon quality in greenhouse farming compared to that in open fields (Figure 5a). We further found the direct effects of cultivation modes and soil protistan compositions on lemon quality (Figure 5d). For the greenhouse practices, several potential reasons could lead to the reduction in lemon quality. Greenhouses are more suitable for planting short-term small plants; in contrast, the lemon tree is a perennial large plant and long-term exposure to greenhouse conditions can lead to a decrease in its vitality [60,61]. In addition, greenhouses generally possess a higher plant density, which increases the competition for light and then influences their growth [62]. For the effects of soil protistan community on lemon quality, our results indicated relatively weak nutrient cycling of protists in greenhouses, which could be driven by the low level on nitrogen in greenhouse farmlands (Figure 1a). In conclusion, open-field cultivation could be considered a more suitable agricultural mode for lemon cultivation.

## 5. Conclusions

The findings of this study demonstrated that the cultivation mode exerted an influence on the soil properties, instigated succession in the soil protistan community, and regulated the production quality in lemon farmlands. In contrast to open-field cultivation, the use of a greenhouses has been demonstrated to engender a decline in the nutrient content and enzyme activity of the soil. This has been shown to result in a reduction in diversity and alteration to the composition of protistan communities. During the growth phase of the lemon, the process of greenhouse farming resulted in an enrichment of Archaeplastida in farmland soils, while Stramenoplies and Alveolata were reduced at the flowering and fruiting stages, respectively. The analysis of functional traits indicated that the greenhouse environment led to an increase in the relative abundance of phototrophs while concurrently resulting in a decrease in consumers within the farmland soils when compared to open-field cultivation. Furthermore, deterministic assembly was demonstrated to govern the succession of soil protistan communities in lemon farmlands, with nitrogen level identified as the dominant driving factor. Finally, the lowest quality of lemons was observed in greenhouse farmlands, which resulted in the direct effects of greenhouse farming and indirect effects mediated by soil protistan communities. The findings of this study have enhanced our comprehension of soil protists in agricultural ecosystems and provide a foundation for the future enhancement of the management of lemon cultivation. The improvement of planting strategies and operational methods in greenhouses to enhance lemon quality represent a future research direction.

## Data Availability

The raw sequencing data for this study can be found in the NCBI SRA database with accessible number PRJNA1063860.

## Supplementary Materials

The following supporting information can be downloaded at: https://www.mdpi.com/article/10.3390/plants14132024/s1, Figure S1: Differences in the soil properties between different cultivation modes and different lemon growth stages. Different lowercases letters in each subfigure represent significant differences between different groups (Tukey’s HSD test, *p* < 0.05); Figure S2: Annotation ratio of protistan ASVs at different taxonomy levels; Figure S3: Rarefaction (a) and species accumulation (b) curves of all groups; Figure S4: Relative abundance (%) of soil protistan species among different farmlands; Figure S5: Differences in the relative abundance of soil protistan species between different cultivation modes and different lemon growth stages. Different lowercases letters in each subfigure represent significant differences between different groups (Tukey’s HSD test, *p* < 0.05). Figure S6: Differences in the lemon quality parameters between different cultivation modes and different lemon growth stages. Different lowercases letters in each subfigure represent significant differences between different groups (Tukey’s HSD test, *p* < 0.05).
