# Supplementary material for: Effects of Cultivation Modes on Soil Protistan Communities and Its Associations with Production Quality in Lemon Farmlands
Source: Plants (Basel). 2025 Jul 2;14(13):2024. doi: 10.3390/plants14132024 (PMC12252243; doi:10.3390/plants14132024)
Supplement: Supplementary file 1 [file plants-14-02024-s001.zip › plants-3709086-supplementary.pdf]

plants

Supplementary Material

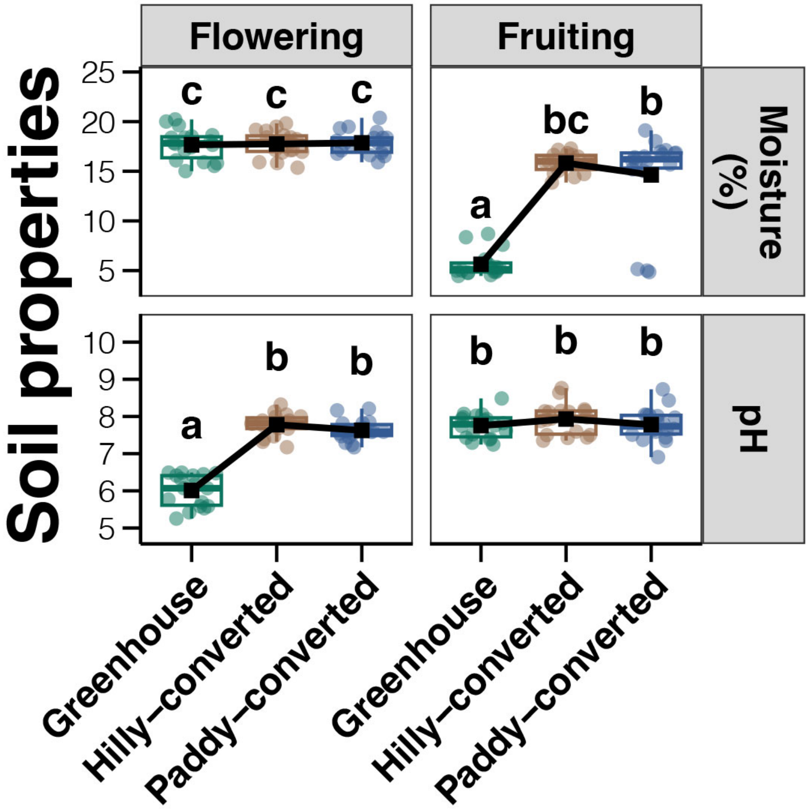

Supplementary Figure S1. Differences in the soil properties between different cultivation modes and different lemon growth stages. Different lowercases letters in each subfigure represent significant differences between different groups (Tukey's HSD test, $p < 0.05$).

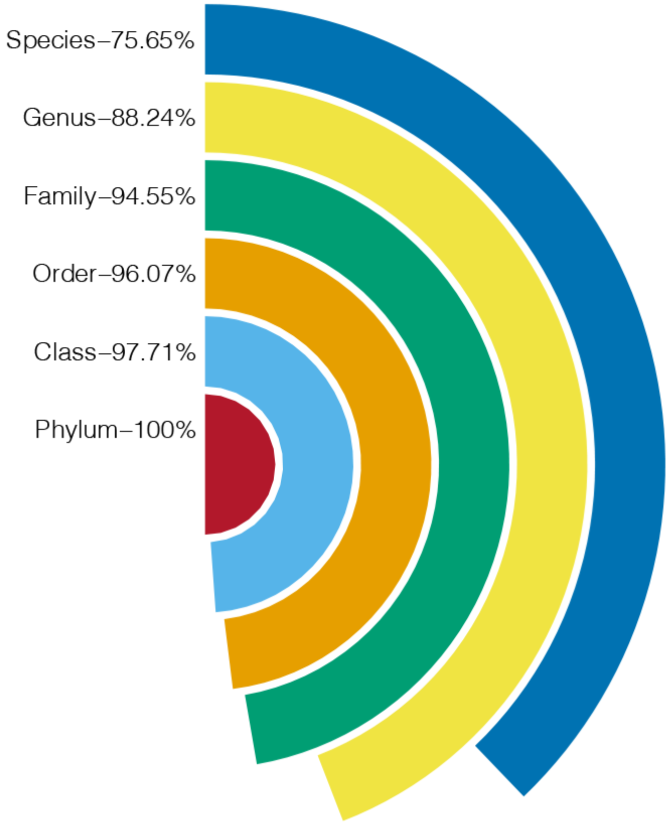

Supplementary Figure S2. Annotation ratio of protistan ASVs at different taxonomy levels.

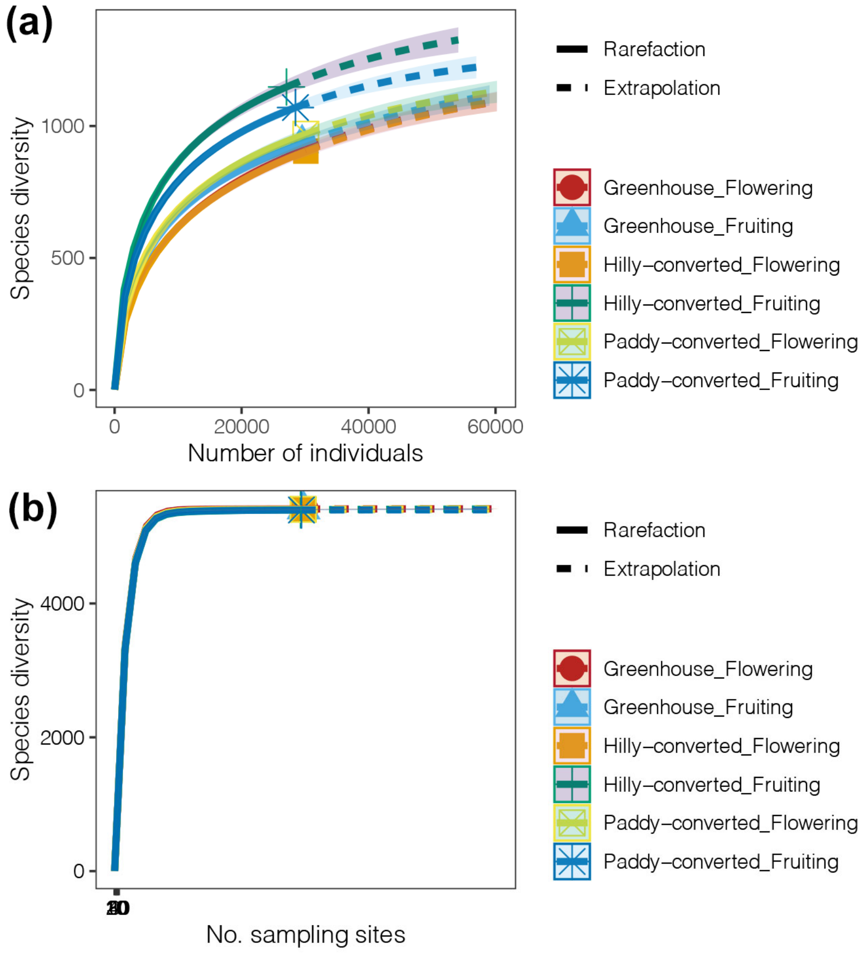

Supplementary Figure S3. Rarefaction (**a**) and species accumulation (**b**) curves of all groups.

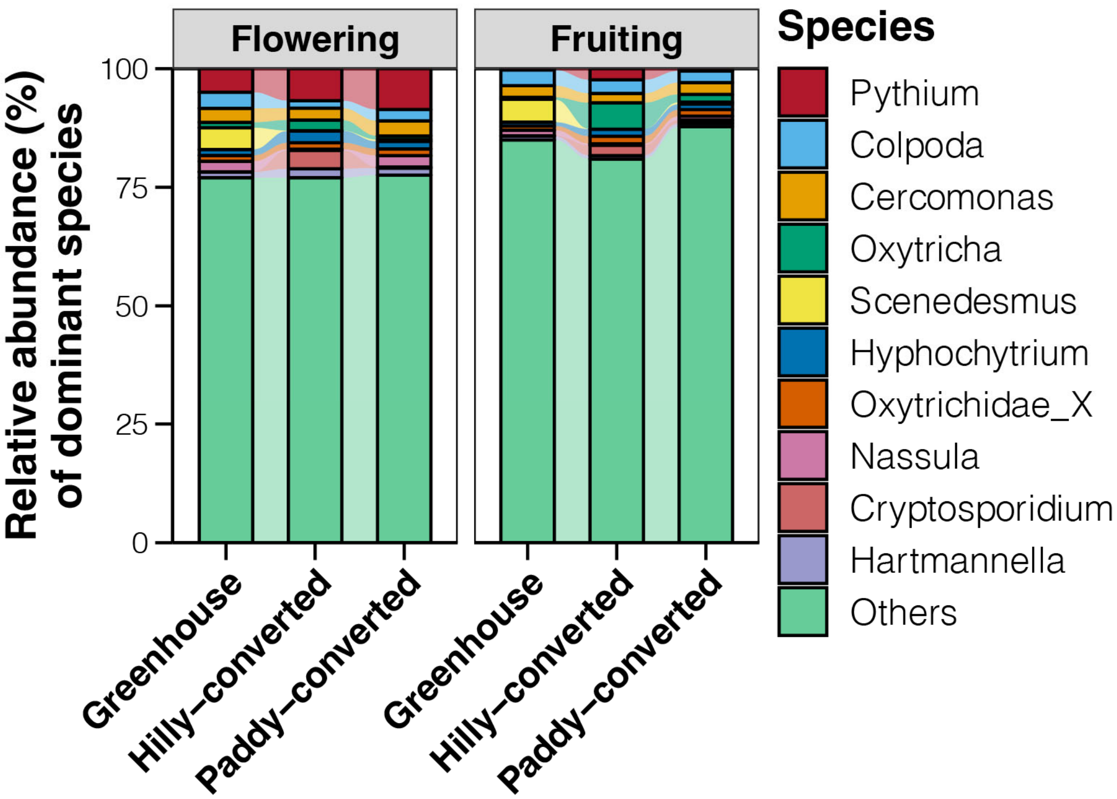

Supplementary Figure S4. Relative abundance (%) of soil protistan species among different farmlands.

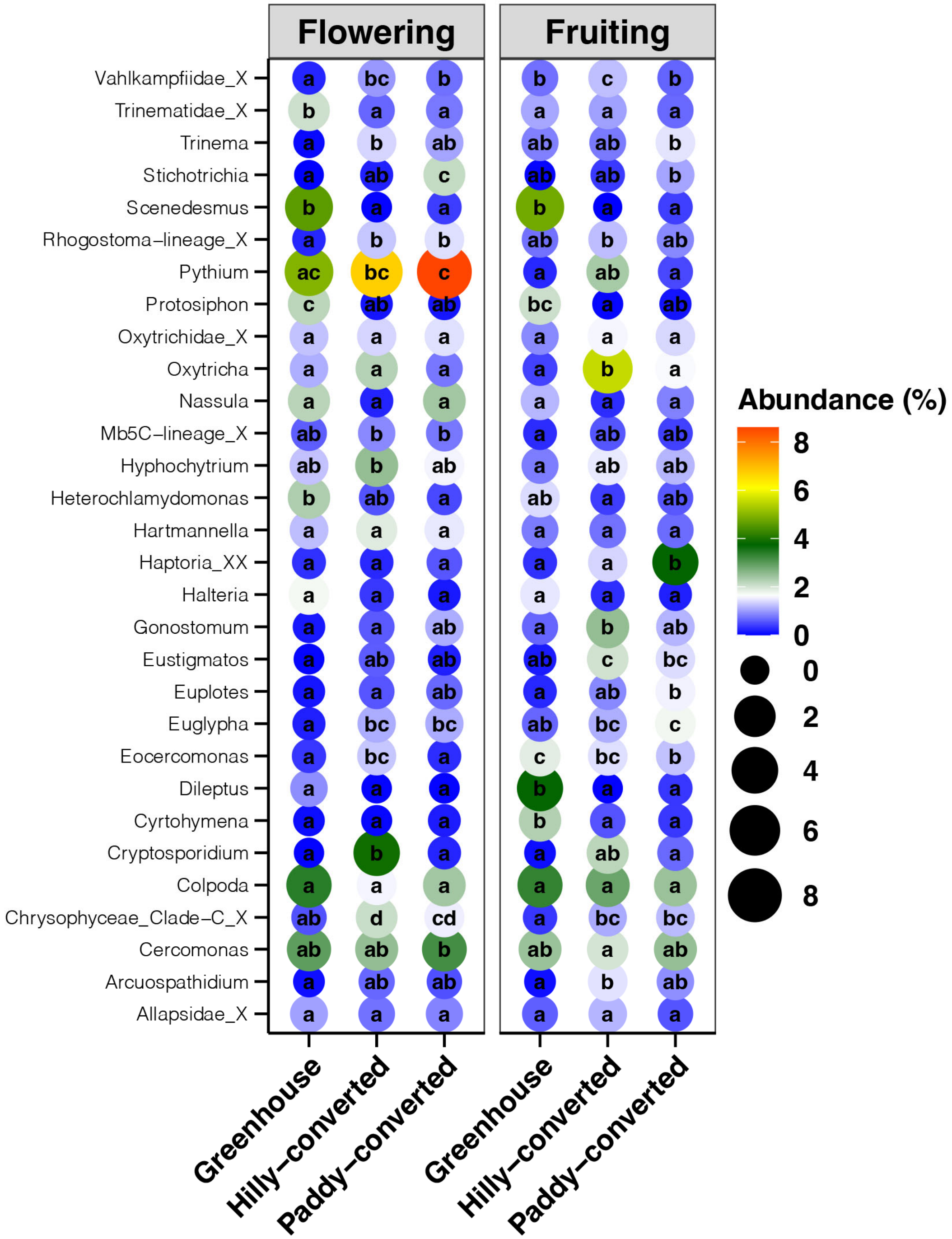

Supplementary Figure S5. Differences in the relative abundance of soil protistan species between different cultivation modes and different lemon growth stages. Different lowercases letters in each subfigure represent significant differences between different groups (Tukey's HSD test, $p < 0.05$).

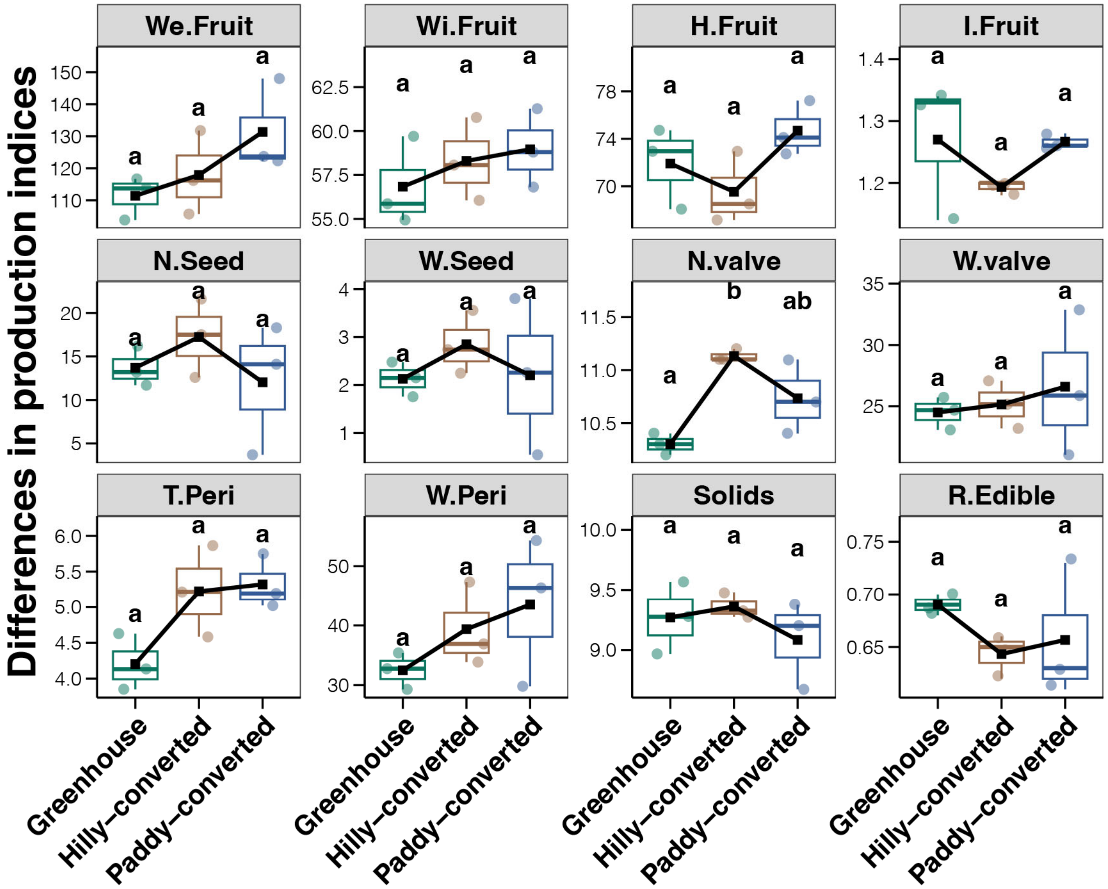

Supplementary Figure S6. Differences in the lemon quality parameters between different cultivation modes and different lemon growth stages. Different lowercases letters in each subfigure represent significant differences between different groups (Tukey's HSD test, $p < 0.05$).
